# Urinary coenzyme Q10 as a diagnostic biomarker and predictor of remission in a patient with ADCK4-associated Glomerulopathy: a case report

**DOI:** 10.1186/s12882-020-02208-7

**Published:** 2021-01-07

**Authors:** Yan Zhang, Xiaohua Liao, Yupeng Jiang, Xin Lv, Yue Yu, Qin Dai, Liyun Ao, Lijian Tao, Zhangzhe Peng

**Affiliations:** Department of Nephrology, Xiangya Hospital, Central South University, Changsha, 410008 Hunan China

**Keywords:** ADCK4 mutation, Coenzyme Q10, Proteinuria, Biomarker, Case report

## Abstract

**Background:**

AarF domain-containing kinase 4 (ADCK4)-associated glomerulopathy is a mitochondrial nephropathy caused by mutations in the ADCK4 gene, which disrupt coenzyme Q10 biosynthesis.

**Case presentation:**

We report the case of a 25-year-old female patient with ADCK4-associated glomerulopathy presenting with proteinuria (and with no additional systemic symptoms). A known missense substitution c.737G > A (p.S246N) and a novel frameshift c.577-600del (p.193-200del) mutation were found. We followed the patient for 24 months during supplementation with coenzyme Q10 (20 mg/kg/d – 30 mg/kg/d) and describe the clinical course. In addition, we measured serum and urine coenzyme Q10 levels before and after coenzyme Q10 supplementation and compared them with those of healthy control subjects. The patient’s urinary coenzyme Q10 to creatinine ratio was higher than that of healthy controls before coenzyme Q10 supplementation, but decreased consistently with proteinuria after coenzyme Q10 supplementation.

**Conclusions:**

Although the use of urinary coenzyme Q10 as a diagnostic biomarker and predictor of clinical remission in patients with ADCK4-associated glomerulopathy should be confirmed by larger studies, we recommend measuring urinary coenzyme Q10 in patients with isolated proteinuria of unknown cause, since it may provide a diagnostic clue to mitochondrial nephropathy.

## Background

Persistent proteinuria is a warning sign of a variety of kidney diseases, often requiring more intensive tests, such as a renal biopsy. Mitochondrial dysfunction due to mutations in genes encoding proteins involved in energy production can lead to kidney diseases [1, 2]. These disorders present clinically as nephrotic syndrome, renal tubular dysfunction and renal dysfunction with proteinuria [3]. As an essential molecule involved in electron transport in the respiratory chain, coenzyme Q10 (CoQ10) plays a crucial role in maintaining normal mitochondrial function. The AarF domain containing kinase 4 (ADCK4) gene is located on human chromosome 19q13.2 and encodes a protein that is involved in the synthesis of CoQ10 [4]. The kidney biopsy often suggests focal segmental glomerulosclerosis (FSGS) in ADCK4-associated glomerulopathy. CoQ10 supplementation could be an effective treatment, and indeed, there are reports of successful treatment of patients with ADCK4 mutations using CoQ10 (Table 1) [4–7].

**Table 1:** Patients with ADCK4 mutations treated with CoQ10 reported to date

Nucleotide mutation	Exon	Amino acid change	Age at onset	Kidney disease	Origin	Histology	Renal ultrasound	CoQ10 start	Treatment/CoQ10 dose (duration)	Response	Extrarenal findings	Reference
c.1199-1200insA	13(hom)	p.H400Nfs∗11	< 1 yr	NS	Turkey	FSGS	NDA	1 yr	CoQ10: 15 mg/kg/d; prednisolone, ACE-I, cyclophosphamide	Proteinuria partial remission	Neurological development delay	[4]
c.293 T > G	NDA	p.L98R	27 yr	Proteinuria	Turkey	FSGS	NDA	27 yr	CoQ10: 10 mg/kg/d	Proteinuria partial remission	None	[5]
c.1339dupG	NDA	p.E447Gfs∗10	9 yr	Albuminuria	Turkey	FSGS	NDA	9 yr	CoQ10: 30 mg/kg/d	Proteinuria partial remission	None	[5]
c.748G > C c.532C > T	9(hom) 7(hom)	p.D250H p.R178W	9 mo	Proteinuria	China	NDA	Normal	9 mo	CoQ10:15–30 mg/kg/d (12mo)	Proteinuria full remission	Mental developmental retardation	[6]
c.614C > T	8(hom)	p.S205N	11 yr	NS	China	FSGS	Echo enhancement	11 yr	CoQ10:15–30 mg/kg/d (12mo)	Progress to CKD	None	[6]
c.625C > G	NDA	p.D209H	14 yr	Proteinuria	China	FSGS	Increased medullary echogenicity	14 yr	CoQ10:150 mg/d (3mo)	uPCR:1.54 g/g to 0.95 g/g	None	[7]

*NS* Nephrotic syndrome, *FSGS* Focal segmental glomerulosclerosis, *ACE-I* Angiotensin-converting-enzyme inhibitors, *NDA* No data available, *CKD* Chronic kidney disease, *uPCR* Urinary protein/creatinine ratio, *hom* Homozygous, *yr* Year-old, *mo* Month

Here, we report the case of a 25-year-old female patient with ADCK4-associated glomerulopathy, with heavy proteinuria, but no systemic symptoms. During a 24-month follow-up, she showed a significant reduction in urinary protein after CoQ10 supplementation.

## Case presentation

A 25-year-old woman was examined at nephrology outpatient clinic due to 3+ proteinuria discovered during a physical examination. She had no history of hematuria, edema, arthralgia, rash or other manifestations. Laboratory tests revealed hypoalbuminemia (35.0 g/L) and elevated blood lipid (high density lipoprotein, 1.88 mmol/L) and creatinine (1.19 mg/dL). Her urinary protein excretion was 2.62 g/24 h. Anti-neutrophil cytoplasmic antibodies, complement C3 and C4, anti-nuclear antibodies and other systemic lupus erythematosus-related tests were negative. Treatment with an angiotensin receptor blocker (ARB) (candesartan, 4–8 mg, orally once daily) was recommended. Three months later, her proteinuria was 4+. At that point, she was admitted to the nephrology department for a kidney biopsy.

The patient denied any history of hypertension, diabetes or viral hepatitis, but had a history of allergic purpura after eating seafood. There were no manifestations of heart or neuromuscular diseases. She was a student who consumed a regular diet and followed a routine in her daily life. Her parents were both healthy, without a history of consanguineous marriages or similar diseases in their families. On physical examination, no positive signs were found.

**Fig. 1:**
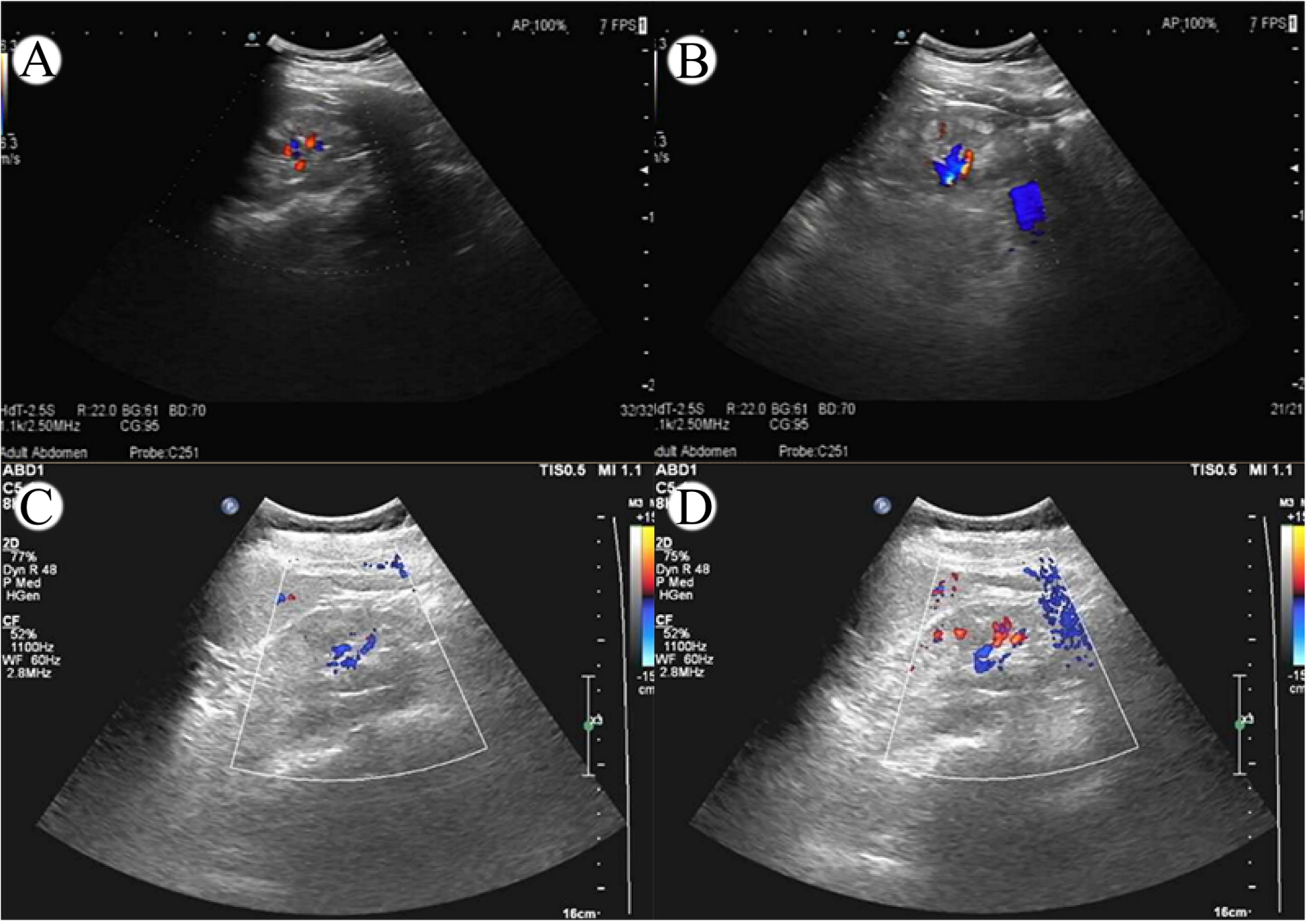
Renal ultrasonography findings in the patient before and after CoQ10 treatment. **a**, **b** Increased echogenicity of renal medullae before treatment with CoQ10. **c**, **d** Normal kidney echogenicity after treatment with CoQ10

After admission, the urinalysis showed proteinuria (protein excretion, 5.39 g/24 h). Urine specific gravity, osmotic pressure, and blood/urine light chain ratio were within the normal range. Evaluation revealed impaired renal function with elevated serum creatinine (1.38 mg/dL) and hypoalbuminemia (32.6 g/l), but without hypercholesterolemia or peripheral edema. The Doppler ultrasound showed a thin renal cortex with bilateral renal vertebral echo enhancement, suggestive of a medullary sponge kidney (Fig. 1 A, B). The left kidney measured 97 × 43 mm, and the right kidney 89 × 35 mm. The patient was not subjected to a renal biopsy due to her small kidneys and thin renal cortices.

The patient underwent genetic analysis and continued taking an ARB (valsartan, 80 mg, orally once daily). Whole exome sequencing demonstrated compound heterozygote mutations in the ADCK4 (CQ8B) gene involving two pathogenic variants: a known missense substitution c.737G > A (p.S246N) and a novel frameshift c.577-600del (p.193-200del) mutation. In addition to ADCK4, we also identified another mutation: a c.2173A > G (p.K725E) mutation in chr2–228,144,556 affecting the COL4A3 gene (Table 2).

**Table 2:** The results of the next-generation sequencing of the patient

Variant gene	Location	Transcriptional exons	Nucleotide amino acids	Homozygous /heterozygous	Prevalence in Control population	Analysis of pathogenicity	Phenotype of mutation	Source
COL43A	chr2–228,144,556	NM-000091; exon29	c.2173A > G (p.K725E)	Het	Not reported	Uncertain	Alport syndrome; familial hematuria	Father
COQ8B	chr19–41,209,508	NM-024876; exon9	c.737G > A (p.S246N)	Het	0.00435	Uncertain	Type 9 nephrotic syndrome	Father
COQ8B	chr19–41,209,736-41,209,778	NM-024876; exon8	c.577-600del (p.193-200del)	Het	Not reported	Uncertain	Type 9 nephrotic syndrome	Mother

Sequencing analysis of the patient demonstrating the detection of c.737G > A (p.S246N) mutation in exon 9 and c.577-600del AGAGTTCTTGAAGAGGAGCTCGGC (p.193-200del) in exon 8 of the COQ8B gene

**Fig. 2:**
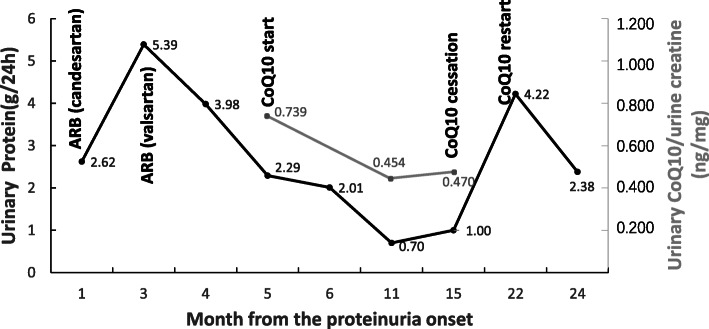
Changes of 24-h urinary protein excretion (g/24 h) and urinary coenzyme Q10 to creatinine ratio in the course of treatment

After genetic diagnosis, the patient began treatment with CoQ10 supplement at a dose of 20 mg/kg per day. After 6 months, her urinary protein decreased significantly from 5.39 g/24 h to 0.70 g/24 h, and her serum creatinine levels remained stable (1.47 mg/dL). The patient did not experience any side effects during the treatment, so we recommended that she continue taking the supplement CoQ10 at a dose of 30 mg/kg per day [5]. After 10 months of treatment with CoQ10 and ARB, her urinary protein remained stable at 1.00 g/24 h. Renal ultrasonography showed normal echogenicity of the kidneys after treatment with CoQ10 (Fig. 1 C, D). However, from then on, the patient only complied with the ARB treatment but did not follow our advice to continue taking CoQ10. Seven months later, her urinary protein rose to 4.22 g/24 h. Based on this result, we recommended her to continue taking the CoQ10 supplement regularly. After another 2 months, her urinary protein decreased from 4.22 g/24 h to 2.38 g/24 h. Glomerular filtration rate remained stable throughout the clinical course. Changes in proteinuria and glomerular filtration rate (eGFR) in the course of treatment are summarized in Fig. 2 and Fig. S1.

We measured the concentration of CoQ10 in the urine of the patient before and after treatment, and corrected it with urinary creatinine. The results showed that the ratio of urinary CoQ10 to creatinine in the patient before treatment (0.739 ng/mg) was nearly 5 times higher than in healthy controls (average value: 0.157 ng/mg, 95% confidence interval: 0.036–0.205) whose eGFR were in normal range and proteinuria were negative. However, after CoQ10 supplementation the ratio decreased (after 6-months: 0.454 ng/mg; after 10-months: 0.470 ng/mg) but was still nearly 3 times higher than in healthy controls (Fig. 2). On the other hand, the serum levels of CoQ10 in the patient before and after treatment were comparable to those of healthy controls.

## Discussion and conclusions

Coenzyme Q10 is a lipid-soluble quinone present in the mitochondrial inner membrane of eukaryotic cells that participates in many important biochemical reactions during electron transport. It modulates metabolism and cell differentiation [8]. To date, 15 genes involved in CoQ10 biosynthesis have been identified in humans. Mutations in eight of them (PDSS1, PDSS2, COQ2, COQ4, COQ6, ADCK3, ADCK4 and COQ9) cause primary CoQ10 deficiency, which result in several disorders [9]. In the first and largest to date multicenter cohort study of Chinese pediatric patients with steroid-resistant nephrotic syndrome (SRNS), ADCK4 was identified as the most common pathogenic gene [10]. So far, only a few studies have described the response of patients with kidney disease due to ADCK4 mutations in an early stage to treatment with CoQ10. All the cases described presented with proteinuria, and the kidney biopsies of most individuals showed FSGS. Other concomitant symptoms which have been described include neurologic developmental delay, mental retardation, occasional seizures and retinitis pigmentosa [4–7].

In this patient the sole clinical manifestation was persistent proteinuria, and genetic testing revealed complex heterozygous ADCK4 (COQ8B) mutations. During the 24-month follow-up, we observed that her proteinuria decreased significantly after taking CoQ10, but worsened after interruption of treatment with CoQ10. Serum creatinine levels in the patient remained stable and no side effects were observed during the follow-up. Based on the response of our patient to CoQ10, we believe that supplementation with oral CoQ10 may reverse proteinuria in adult-onset ADCK4-associated glomerulopathy, not only in early or adolescent-onset patients.

The only available method to confirm the diagnosis of ADCK4-associated glomerulopathy is genetic testing, but this cannot be carried out routinely because of its high cost and the length of time needed for completion. This often leads to missed diagnosis and misdiagnosis. CoQ10 levels can be measured in different specimens, such as plasma, serum, blood mononuclear cells, platelets, urine, muscle and cultured skin fibroblasts [11]. We found that the ratio of urinary CoQ10 to creatinine in our patient before treatment was nearly 5 times higher than in healthy controls. Following the treatment with CoQ10, there was a marked decrease in urinary CoQ10, which was consistent with proteinuria remission. Interestingly, this phenomenon is consistent with the change of plasma CoQ10 in a patient with proteinuria caused by a COQ6 gene defect [12]. The decrease of urinary CoQ10 concentration in our patient during therapy is unclear. In general the urinary CoQ10 concentration is mainly affected by de novo synthesis [13]. Perhaps other tissues may have some compensatory effect for the de novo synthesis of CoQ10, which makes urinary CoQ10 in patient with ADCK4-associated glomerulopathy higher than that of healthy controls. This compensatory effect was weakened after exogenous supplementation with CoQ10, resulting in a decrease in urinary coenzyme q10. Hence, noninvasive evaluation of urinary CoQ10 may be useful and convenient for the diagnosis of ADCK4-associated glomerulopathy and for monitoring the effects of treatment.

It is also worth noting that the patient’s renal color Doppler ultrasound suggested a “medullary sponge kidney” (Fig. 1 A, B) and that her renal medullae returned to normal after treatment with CoQ10 (Fig. 1 C, D). In 2017, it was reported that renal ultrasound examination of 7 patients with ADCK4 mutations showed “enhanced echogenicity of the renal medulla”, which is considered as medullary nephrocalcinosis, but that no related manifestations of medullary sponge kidney were found [14]. In addition, a few studies also reported increased medullary echogenicity in ADCK4-associated glomerulopathy [6, 7, 15]. Whether there is a correlation between renal medullary lesions detected by ultrasound and ADCK4-associated kidney disease remains to be explored. This could be another useful clinical diagnostic sign of mitochondrial nephropathy.

In conclusion, ADCK4-associated glomerulopathy should be considered not only in children but also in young adults with isolated proteinuria of unknown cause. Considering that genetic testing is expensive and time-consuming, the ratio of urinary CoQ10 to creatinine may be a suitable diagnostic biomarker and a predictor of remission in patients with primary coenzyme Q10 deficiency of the kidney. Patients with an elevated ratio of urinary CoQ10 to creatinine and increased medullary echogenicity can be treated empirically with a CoQ10 supplement. Although the clinical response to CoQ10 was evident in our patient, more information is needed about its effectiveness in order to determine when to suspect ADCK4-associated glomerulopathy and which patients with proteinuria should be treated with CoQ10.

## Supplementary Information

**Additional file 1: Fig. S1** Patient’s glomerular filtration rate (eGFR) (mL/min) during a 22 months follow-up.

## Data Availability

Additional data used/generated that is not present in the manuscript is available from the corresponding author upon reasonable request.

## Abbreviations

ADCK4: AarF domain-containing kinase 4
ARB: Angiotensin receptor blocker
CoQ10: Coenzyme Q10
FSGS: Focal segmental glomerulosclerosis
SRNS: Steroid-resistant nephrotic syndrome
