# Supplementary figures and images for: Urinary coenzyme Q10 as a diagnostic biomarker and predictor of remission in a patient with ADCK4-associated Glomerulopathy: a case report
Source: BMC Nephrol. 2021 Jan 7;22:11. doi: 10.1186/s12882-020-02208-7 (PMC7791994; doi:10.1186/s12882-020-02208-7)

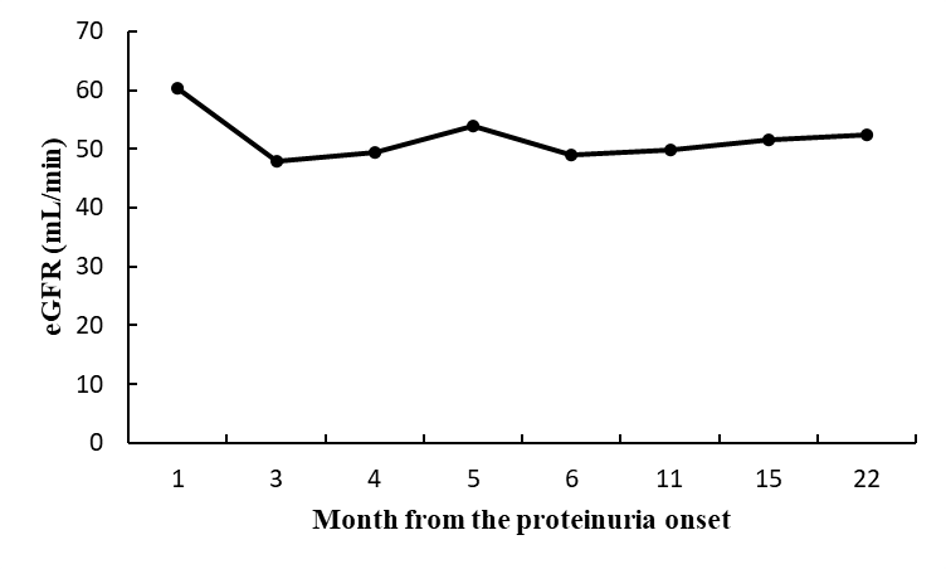

Supplement: Supplementary file 1 — Additional file 1: Fig. S1 Patient’s glomerular filtration rate (eGFR) (mL/min) during a 22 months follow-up. [file 12882_2020_2208_MOESM1_ESM.png]
